# Social Cognitive Predictors of Breakfast Consumption in Primary School’s Male Students

**DOI:** 10.5539/gjhs.v8n1p124

**Published:** 2015-05-15

**Authors:** Amin Mirzaei, Fazlollah Ghofranipour, Zeinab Ghazanfari

**Affiliations:** 1 Department of Health Education, Faculty of Medical Sciences, Tarbiat Modares University, Tehran, Iran; 2 Department of Public Health, Faculty of Health, Ilam University of Medical Sciences, Ilam, Iran

**Keywords:** breakfast consumption, social cognitive theory, primary male students

## Abstract

**Purpose:**

This study aimed to test the usefulness of social cognitive theory (SCT) in explaining breakfast consumption in a sample of primary male students.

**Methods:**

Participants in this cross-sectional study were 358 male students (3rd, 4th and 5th grades) from eight public primary schools of Ilam city. Data were collected by a self-administered questionnaire based on components of SCT. Bivariate correlations and multiple logistic regression analysis using an Enter method were used to identify social cognitive correlates and determinants of breakfast consumption.

**Results:**

A total of 358 participants ranging in age from 8-12 years (M = 10.06) were studied. The result of the study showed that the SCT significantly predicted breakfast consumption. SCT variables explained 41.4% of the variance in breakfast consumption behaviors, though, self-regulation was found to be the strongest predictor of breakfast consumption behaviors. There was the strongest correlation between behaviors and self-regulation, (r=0.561; P <0.001).

**Conclusion:**

The findings support the usefulness of SCT in explaining breakfast consumption behaviors. These results suggest an essential role for self-regulation, self-efficacy and social support in the breakfast consumption behaviors of primary male students.

## 1. Introduction

Breakfast is often called the most important meal of the day (Senanayake & Parakramadasa, 2008; Shaw, 1998). Breakfast consumption after the long period of fasting during the night is important to maintain health, especially during early development in life (Pivik, Dykman, Tennal, & Gu, 2006; Raaijmakers, Bessems, Kremers, & van Assema, 2009). Breakfast consumption has been shown to result in nutritional profile improvements among schoolchildren (Cheng, Tse, Yu, & Griffiths, 2008; Nicklas, Reger, Myers, & O’Neil, 2000; Tin, Ho, Mak, Wan, & Lam, 2011). Eating breakfast can impact favorably on children’s health and well-being, since it is related to higher overall diet quality, nutritional status, participation in physical activities, decreased consumption of snacks and larger meal portions throughout the day as well as decreased risk of overweight (Levin, Kirby, & Currie, 2012; Raaijmakers et al., 2009; Tin et al., 2011; Yang, Wang, Hsieh, & Chen, 2006). In addition, eating breakfast has been found to be associated with higher improvement of mental powers, concentration, cognitive and academic performance (Lopez-Sobaler, Ortega, Quintas, Navia, & Requejo, 2003; Powell, Walker, Chang, & Grantham-McGregor, 1998; Vaisman, Voet, Akivis, & Vakil, 1996).

Despite the positive effects of consuming a healthy breakfast, a large number of children do not frequently eat breakfast. According to a recent review of studies on breakfast consumption patterns around the world reported, published rates of breakfast skipping variety from 1.7% in Croatia to 30% in Brazil (Kothe, Mullan, & Amaratunga, 2011; Mullan & Singh, 2010). The skipping breakfast in Iranian schoolchildren ranged from 4.4% in Semnan to 21% in Qom (Chapman, Melton, & Hammond, 1997; Sheeshka, Woolcott, & MacKinnon, 1993). No studies have been done in this topic in the Ilam city.

The dietary intake patterns of children have been a special concern because it was found that eating patterns formed in early life are likely to continue into adulthood (Chitra & Reddy, 2007; Pearson, Biddle, & Gorely, 2009). Before planning and developing of interventions to promote the breakfast consumption in schoolchildren, clarify the personal and motivational factors behind regular breakfast consumption is necessary.

There are a number of social-cognitive models that attempt to understand these factors behind behavior. Bandura’s Social Cognitive Theory (SCT) delineates the supposed sources and mediators of behavior and behavior change. SCT explains how people attain and maintain definite behavioral patterns, for example eating behaviors (Anderson, Winett, & Wojcik, 2007; Reynolds, Hinton, Shewchuk, & Hickey, 1999; Van Zundert, Nijhof, & Engels, 2009). SCT suggest that behavior, personal factors, and environmental factors interact to explain and predict changes in behavior. Bandura called these relationships “reciprocal determinism”, meaning that, as the three components interact, a change in one will produce changes in the others (Anderson, Winett, Wojcik, Winett, & Bowden, 2001; Glanz, Rimer, & Viswanath, 2008; Reynolds et al., 1999). Environmental influences, such as social support (The perceived support for healthy behavior from important others, for example family and friends), create opportunities and provide reinforcement for behavior change. Personal factors, such as perceived self-efficacy (belief in my ability to change a behavior), outcome expectations (the perceived costs and benefits of the behavior) and outcome expectancies (perceived importance of the potential costs and benefits of the behavior) provide direct causal influences on behavior and are used to interpret information from the environment. Also, self-regulatory behavior (especially goal setting) has been associated with health behavior as healthier eating (Anderson, Winett, & Wojcik, 2000; Anderson et al., 2001; Bandura, 1997; Pelletier, Dion, Slovinec-D’Angelo, & Reid, 2004).

Previous studies showed that SCT has been used in predicting and explaining eating behaviors in adults and is well suited to studies of children’s eating behaviors (Lewis, Sims, & Shannon, 1989; Perry et al., 1990). But no any previous study has ever used the SCT constructs to predicting and explaining the breakfast consumption in schoolchildren. Therefore, the aim of present study was to test the usefulness of SCT in explaining breakfast consumption in a sample of male students from primary schools.

## 2. Materials and Methods

### 2.1 The study and Participants

This cross-sectional study was carried out in Ilam city (west of Iran) between January and December 2013-2014. Participants were 358 male schoolchildren aged 8-12 years (3rd, 4th and 5th grades) from eight primary schools of Ilam city, so that Ilam city divided to four educational areas, and two public schools from each area were selected. Being in 3rd, 4th and 5th grades and satisfy to participate in study were the main inclusion criteria. Research Ethics Committee of Tarbiat Modares University (TMU) approved this study.

### 2.2 Measures

The present study used a researcher-made questionnaire based on SCT. The questionnaire includes three sections: Socio-demographic characteristics such as students’ age and grade; parents’ education, age, occupation and social-economical status (SES).

Social Cognitive Theory (SCT) Scale included six subscales for assessments of knowledge, outcome expectations, outcome expectancies, self-efficacy, social support and self-regulation. The knowledge subscale included 6 multiple-choice items that measured the knowledge needed to enact the breakfast consumption behaviors. Example item; *It* *is the most important meal of the day*. Outcome expectations and outcome expectancies subscales included 16 items (8 corresponding items for each construct). These subscales were rated on a 5-point likert scale (1 = strongly disagree to 5 = strongly agree). Outcome expectations example item; *If I eat breakfast, I will have healthy body*. Outcome expectancies example item; *It is important for me to have a healthy body*. The self-efficacy subscale was rated on a 5-point likert scale (1 = strongly disagree to 5 = strongly agree) and included 7 items. Example item; *I am confident that I can eat breakfast, even when I wake up late*. Also, social support subscale included 8 items that was rated on a 5-point likert scale (1 = strongly disagree to 5 = strongly agree), Example item; *My family encourage me to eat breakfast*. And self-regulation subscale was rated on a 5-point likert scale (1 = never to 5 = always), included 11 items. Example item; *I decided to eat breakfast before sleeping at night*.

Behavior scale include 9 items that was rated on an 8-point Likert scale (from never to 7 day), Example item; *During the past 7 days, how often did you eat breakfast?*

Internal consistency (Kuder Richardson-20 and Cronbach’s alpha) of scales showed in Table 1. Time needed to complete the questionnaire was approximately 25 minutes.

**Table 1:** Internal consistency estimates of social cognitive measures and breakfast consumption behaviors

Measures	No. of Items	Internal Consistency
Knowledge	6	0.62^a^
Outcome expectations	8	0.90^b^
Outcome expectancies	8	0.89^b^
Self-efficacy	7	0.86^b^
Social support	8	0.65^b^
Self-regulation	11	0.86^b^
Behaviors	9	0.90^b^

a Kuder Richardson-20 (KR-20),

b Cronbach’s alpha (α).

### 2.3 Statistical Analysis

Descriptive statistics, bivariate correlations, and multiple logistic regression analysis (Enter method) were calculated using SPSS-16. P-values less than 0.05 were considered to be statistically significant.

## 3. Results

Data was collected from 358 primary school’s male students ranged from 8 to 12 years (M = 10.06). The socio-demographic characteristics of subjects are presented in Table 2.

**Table 2:** Socio-demographic characteristics of study subjects

Characteristics	N	%
**Age**		
8	7	2.0
9	100	27.9
10	130	36.3
11	107	29.9
12	14	3.9
**Students Educational Grades**		
3rd	97	27.1
4th	130	36.3
5th	131	36.6
**Father educational level**		
Illiterate	12	3.4
High school and less than	140	39.1
Diploma or higher	206	57.5
**Mother educational level**		
Illiterate	20	5.6
High school and less than	162	45.3
Diploma or higher	176	49.2
**Father occupational situation**		
Unemployed	10	2.8
Laborer	50	14.0
Employee	135	37.7
Self employment	156	43.6
Dead	7	2.0
**Mother occupational situation**		
Householder	327	91.3
Laborer	1	0.3
Employee	16	4.5
Self employment	14	3.9
**Socioeconomic status (SES)**		
Low	93	26.0
Intermediate	224	62.6
High	41	11.5

Means, standard deviations and inter-correlations of social cognitive measures and behavior had shown in Table 3. As shown, knowledge, outcome expectations, outcome expectancies, self-efficacy, social support, and self-regulation measures were all significantly correlated with breakfast consumption behaviors. There was the strongest correlation between behaviors and self-regulation, (r=0.561; P < 0.001).

**Table 3:** Means, standard deviations, and inter-correlations for breakfast consumption behaviors and social cognitive variables

Variables	1	2	3	4	5	6	7	M	SD
1.Knowledge	1							4.38	1.17
2.Outcome expectations	.269*	1						33.93	4.06
3.Outcome expectancies	.348*	.614*	1					34.76	3.72
4.Self-efficacy	.266*	.507*	.466*	1				29.81	4.50
5.Social support	.280*	.425*	.412*	.595*	1			30.67	4.26
6.Self-regulation	.258*	.394*	.291*	.376*	.453*	1		37.78	7.16
7.Behaviors	.280*	.409*	.380*	.452*	.459*	.561*	1	42.56	9.08

* Correlation is significant at the 0.01 level (2-tailed).

A multiple regression analysis was carried out to examine the predictive power of SCT structures in breakfast consumption behaviors. For this purpose, SCT variables were entered into a multiple regression analysis to evaluate their exclusive contribution to predicting behaviors. The overall model was significant (R2=0.414; F=41.27, P<0.001). All SCT variables simultaneously explained 41.4% of the variance in breakfast consumption behaviors.

Only self-efficacy, social support, and self-regulation were significant predictors of behaviors, but knowledge, outcome expectations and outcome expectancies were not significant. Self-regulation was the strongest predictor of behaviors and singly explained the 31.5% of the variance in behaviors (Table 4).

**Table 4:** Multiple regression analysis: Variables predicting breakfast consumption behaviors

	B	β	R	R2	F	Sig.
Knowledge	.459	**.059**				.183
Outcome expectations	.132	**.059**				.294
Outcome expectancies	.240	**.098**				.073
Self-efficacy	.305	.151				.006**
Social support	.238	.112				. 040*
Self-regulation	.487	.384	.644	.414	41.270	. 000**

* Significant at the 0.05 level (2-tailed),

** Significant at the 0.01 level (2-tailed).

N = 358; dependent variable: Breakfast consumption behaviors.

Whereas, the self-regulation was the most important predictor of breakfast consumption behaviors, other SCT variables were entered in the regression analyses model to examine the power of these variables in explaining of self-regulation in the single step. As revealed in Table 5, the overall model was significant (R2=0.269; F=25.87, P < 0.001). All five variables together accounted for 26.9% of the variance in self-regulation. But, merely knowledge, outcome expectations and social support were significant predictors of self-regulation.

**Table 5:** Multiple regression analysis: Variables predicting self-regulation

	B	β	R	R^2^	F	Sig.
Knowledge	.654	.107				.030*
Outcome expectations	.402	.228				.000**
Outcome expectancies	-.078	-.040				.510
Self-efficacy	.122	.077				.212
Social support	.497	.296	.519	.269	25.870	.000**

* Significant at the 0.05 level (2-tailed),

** Significant at the 0.01 level (2-tailed).

N = 358; dependent variable: Self-regulation.

## 4. Discussion

The aim of the present study was to examine the utility of SCT in predicting breakfast consumption behaviors in a sample of male schoolchildren. The findings of this study showed that six SCT variables used in the study simultaneously explained 41.4% of the variance in breakfast consumption behaviors. To the best of our knowledge, there was no study that has been applied SCT to predict breakfast consumption. But, the results of other studies indicated that the SCT explained the nutritional behaviors differently. Reynolds et al. (1999) and Anderson et al. (2007) reported that SCT constructs explained 11% and 61% of variance in fruit and vegetables consumption, respectively (Anderson et al., 2007; Reynolds et al., 1999). So, in similar studies that used the Theory of Planned Behavior (TPB) framework to explaining student’s breakfast consumption, TPB has been varied explaining power in intentions to breakfast consumption range from 39% to 61 % (Kothe et al., 2011; Wong & Mullan, 2009). Only self-regulation, self-efficacy and social support were found to significantly predict breakfast consumption, but knowledge, outcome expectations and outcome expectancies did not. However, all constructs positively correlated with breakfast consumption behaviors. On the basis of present study results, self-regulation had the higher correlation with breakfast consumption and was the strongest predictor of behaviors. Thereafter, self-efficacy and social support were the strongest predictors to breakfast consumption behaviors, respectively. In Anderson et al. (2007) study, self-regulation was the strongest predictor of nutritional behaviors and also, self-efficacy and social support were the important determinants of nutritional behaviors (Anderson et al., 2007). Although, few studies investigated the role of self-regulation in relation to nutritional behaviors, but they have shown the independent self-regulation effects on behavior in SCT, and sometimes mediating the other variables such as social support (Anderson et al., 2007; Bandura, 1997). Higher levels of self-regulation lead individuals to set goals for and plan and monitor their healthier eating behaviors (Bandura, 1991, 2001).

The results indicated the positive significant inter-correlation between three predictor constructs (self-regulation, self-efficacy and social support) of breakfast consumption behaviors. The self-regulation levels in schoolchildren and applying self-regulation strategies to perform the healthy behaviors is in relation to social support and self-efficacy. Therefore, addressing these behavioral mediators together is a vital matter to promote the nutritional behaviors via educational interventions. Despite the independent effects of self-regulation on the nutritional behaviors in adults, using the self-regulation strategies in schoolchildren requires the social support especially family support (Rasmussen et al., 2006).

Results of several studies confirm the present study findings. Lubans et al. (2007) stated that self-efficacy is the main determinant of behavioral intention. In their study self-efficacy had the strongest correlation with nutrition intake in students, whiles self-regulation construct had not been investigated (Lubans et al., 2012). Other studies acquired similar findings (Ball et al., 2009; Tavakoli & Fallahi, 2011; Pearson, Ball, & Crawford, 2011; Rasmussen et al., 2006). Higher levels of self-efficacy increase the capability of school children to utilizing self-regulation strategies to achievement the healthy nutritional behaviors (Anderson et al., 2007; Bandura, 1997, 2004). Also, students perceived social support affect their perceived self-efficacy. In addition, students’ social support specially perceived family support have a strong direct relation with nutritional behaviors. So, as mentioned above, self-regulation and self-efficacy mediate the effect of social support on nutritional behaviors in school children (Anderson et al., 2007; Bandura, 1997, 2004). Bandura has assigned this interaction between personal factors, environmental factors, and behavior in philosophy of SCT (Anderson et al., 2001; Glanz et al., 2008; Lubans et al., 2012; Reynolds et al., 1999). Because of extra effect of environmental factors on schoolchildren nutritional behaviors, planning of interventions must be attend to effective social environment on children behaviors. Engagement of parents and teachers in the interventions is more effective than education of students solely.

Though, knowledge, outcome expectations and outcome expectancies were not significant predictors of breakfast consumption behaviors, but all of these constructs were significantly correlated with the behaviors. School nutrition program as a part of school health programs has been performed in urban primary schools of Ilam. But, most of the educational programs in nutritional issues have been concentrated on increasing the students’ knowledge and benefits of healthy eating such as breakfast consumption. Furthermore, all of these programs have been carried out by inexperienced school health trainers and school teachers. Another matter is the pivotal role of students in these programs. Whiles, we expected that it should be paid more attention to social and environmental determinants of schoolchildren nutritional habits. With regard to abovementioned interpretations, the present results obtained from regression analysis, were rational.

According to the results of present study, knowledge, outcome expectations and social support were significantly predicted self-regulation. The effect of knowledge on the students breakfast consumption behaviors have been reported in several studies. But in many cases this influence is indirect, and inadequate to create or change in nutritional behaviors of pupils (Foley, Vaden, Newell, & Dayton, 1983; Lewis et al., 1989; Reynolds et al., 1999). Even though, increasing the knowledge of schoolchildren and educate them about the benefits and positive outcomes of eating behaviors such as eating breakfast is needed but not sufficient.

On the basis of SCT, schoolchildren should be empowered for performing the health behaviors and using the self-regulation strategies, and they should be supported by family, teachers and friends (Anderson et al., 2007; Bandura, 1997, 2004). The role of family support in children’s eating behaviors has been investigated in several studies (DeJong, van Lenthe, van der Horst, & Oenema, 2009; Nicklas et al., 2001; Patrick & Nicklas, 2005). These studies report that parents play a direct role in shaping the children’s eating patterns through their behaviors, attitudes, and eating styles, including eating breakfast. Several other studies have also reported that parent’s role model has a strong relationship with the schoolchildren eating breakfast (DeJong et al., 2009; Nicklas et al., 2001; Patrick & Nicklas, 2005). Additionally, a supportive home environment, and providing family rules which are largely created by parents, is related with children breakfast consumption both directly and indirectly (DeJong et al., 2009). Thus, addressing parents in future interventions, aimed at improving schoolchildren breakfast consumption is crucial.

### 4.1 Strengths and Limitations

This study is the first study to apply the Bandura’s Social Cognitive Theory framework to explain the breakfast consumption behaviors of primary male students. However, there are limitations to the present study. First, only students in grades third, fourth and fifth were studied, but first and second graders were not investigated because of the student’s inability to read and answer the questions. Second limitation was the trust to self-report measures to collecting data. Although the use of self-reporting is a common approach for collecting data on behavior, Hopefully in future studies, other methods such as observation and recording of children’s eating behavior by others, to be used.

### 4.2 Conclusions

The results of present study provide support for the SCT as a framework to predict the breakfast consumption behaviors. These results suggest an essential role for self-regulation, self-efficacy, and social support in the breakfast consumption behaviors of primary male students. Health education interventions that are effective at acquiring social support, and at increasing self-efficacy, should be more successful at helping schoolchildren perform the self-regulatory behaviors necessary to eating healthy breakfast.
